# Failure of Immunotherapy—The Molecular and Immunological Origin of Immunotherapy Resistance in Lung Cancer

**DOI:** 10.3390/ijms22169030

**Published:** 2021-08-21

**Authors:** Justyna Błach, Kamila Wojas-Krawczyk, Marcin Nicoś, Paweł Krawczyk

**Affiliations:** 1 Department of Clinical Immunology, Medical University of Lublin, W. Chodźki 4A, 20-093 Lublin, Poland; 2 Department of Clinical Immunology, University Children Hospital of Cracow, 30-663 Cracow, Poland; 3 Department of Pneumonology, Oncology and Allergology, Medical University of Lublin, Jaczeswskiego 8, 20-954 Lublin, Poland; kamilawojas@wp.pl (K.W.-K.); marcin_nicos@interia.pl (M.N.); krapa@poczta.onet.pl (P.K.)

**Keywords:** immunotherapy, tumor resistance, immune checkpoint inhibitors, lung cancer, tumor immune escape

## Abstract

Immune checkpoint inhibitors (ICIs) have a huge impact on clinical treatment results in non-small cell lung cancer (NSCLC). Blocking antibodies targeting programmed cell death protein 1 (PD-1), programmed cell death protein ligand 1 (PD-L1) or CTLA-4 (cytotoxic T cell antigen 4) have been developed and approved for the treatment of NSCLC patients. However, a large number of patients develop resistance to this type of treatment. Primary and secondary immunotherapy resistance are distinguished. No solid biomarkers are available that are appropriate to predict the unique sensitivity to immunotherapy. Knowledge of predictive markers involved in treatment resistance is fundamental for planning of new treatment combinations. Scientists focused research on the use of immunotherapy as an essential treatment in combination with other therapy strategies, which could increase cancer immunogenicity by generating tumor cells death and new antigen release as well as by targeting other immune checkpoints and tumor microenvironment. In the present review, we summarize the current knowledge of molecular bases underlying immunotherapy resistance and discuss the capabilities and the reason of different therapeutic combinations.

## 1. Introduction

Cancer is the second cause of death worldwide, with lung cancer at the forefront [1]. Research into effective treatments for lung cancer has been going on for many years. Immunotherapy in non-small cell lung cancer (NSCLC) uses immune checkpoint inhibitors (ICIs), which specifically inhibit T-cell anergy and apoptosis. ICIs are directed against immune checkpoints such as the cytotoxic T lymphocyte antigen 4 (CTLA-4), the programmed cell death 1 (PD-1) and the programmed cell death ligand 1 (PD-L1). Anti-CTLA-4 antibody (ipilimumab) was the first ICI used in cancer therapy, nevertheless, has higher toxicity and less effectiveness compared to anti-PD-1 or anti-PD-L1 inhibitors. Nivolumab and pembrolizumab (anti-PD-1 antibodies) as well as atezolizumab and durvalumab (anti-PD-L1 antibody) are used in monotherapy in the first- and the second-line therapy in advanced NSCLC patients. Combination therapies containing immunotherapy are also used in patients with NSCLC. Pembrolizumab in combination with chemotherapy and nivolumab in combination with ipilimumab or with ipilimumab and chemotherapy are used in first-line treatment. Whereas durvalumab is used in patients with locally advanced NSCLC as consolidation therapy after concurrent chemoradiotherapy [2]. In NSCLC patients, the only validated predictive factors for immunotherapy are: PD-L1 expression on tumor cells, tumor mutations burden (TMB) and microsatellite instability (MSI). However, these predictive factors are not ideal [3]. ICIs are also used in small cell lung cancer (SCLC). FDA approved the use of nivolumab or pembrolizumab as a third-line therapy for metastatic SCLC in 2018. Afterwards, atezolizumab with carboplatin and etoposide and durvalumab in combination with chemotherapy in first-line therapy for extensive disease SCLC (SD-SCLC) were approved in 2020 [4].

Thanks to immunotherapy, it was possible to achieve long-term survival in patients with lung cancer. In the KEYNOTE-001 study with pembrolizumab, five-year overall survival (OS) rate was 23.2% for treatment-naive patients and 15.5% for previously treated patients. In patients with a PD-L1 expression on 50% or greater tumor cells, five-year OS rate was 29.6% and 25.0% in treatment-naive and previously treated patients, respectively [5]. A relatively high percentage of patients with survival over five years was also found in studies on the effectiveness of nivolumab in the second or next-line therapy. In CheckMate 017 and 057 studies, percentage of such patients was 13.4%, whereas in CA209-003—15.6% [6,7]. Immunotherapy turned out to be a breakthrough in NSCLC treatment, but this delight did not last long. It was found that tumors can develop resistance against the immunotherapy. There are three main groups of patients: those who respond initially and still show disease control (long-term responders), those who have never responded (primary resistance), and those who respond initially but eventually develop disease progression (secondary resistance). Around 40–50% of lung cancer patients manifested rapid progression and even hyperprogression during first cycles of immunotherapy. Various mechanisms of resistance to ICIs have been described, including the changes in tumor microenvironment (TME) and mutations in the cancer genome [8,9]. Predictive biomarkers for immunotherapy resistance are important for maximizing the therapeutic effect and testing new therapeutic options including combined treatment. It would be significant to discover the optimal combinations of different types of immunotherapy or immunotherapy with chemotherapy or radiotherapy to overcome drug resistance.

The aim of this study was to review causes of resistance on immunotherapy in lung cancer. The review includes also a discussion about methods of treatment aimed at overcoming resistance to the ICIs and provide future potential perspectives.

## 2. Resistance to Immunotherapy Associated with an Impaired Function of the Immune System

The anti-tumor immune response is an extremely complex multi-stage process depending on many factors. Based on the presence or absence of the immune system in the tumor microenvironment, we can distinguish three immunophenotypes of tumors: (1) “Hot” tumors, which are strongly infiltrated by T lymphocytes and many inflammatory signals are presented; (2) “cold” tumors, which are scanted of any immune cells infiltration nor inflammatory signs; (3) tumors with immune exclusion, where immune cells are at the periphery or within the stromal tissue [10,11,12]. It is postulated that high density of T lymphocytes and pre-existing of primed immune response in “hot” tumors are associated with higher clinical benefits from immunotherapy [10,11,12,13,14,15]. The immunotherapy resistance associated with an impaired function of the immune system, also referred in the literature as tumor extrinsic mechanism, could be considered on several levels of immune system dysfunction and could be associated with specific tumor immunophenotypes [8,16,17].

One of the described mechanisms is related to the lack of specific T lymphocytes in the tumor microenvironment. In addition, T cells, if they are present in the tumor tissue, does not expressed the TCR molecule specific for the tumor antigen. This situation occurs when the mechanisms of antigen presentation are disturbed, and it may occur on many levels. Malfunctioning of antigen presenting cells that are unable to recognize and phagocytose tumor antigens results in failure to elicit a specific immune response. This situation most often takes place in “cold” tumors and is usually associated with primary resistance to immunotherapy [10,14].

Another clinical situation is resistance due to the lack of infiltration of activated T cells into the neoplastic tissue. Although T cells receive activation signals in the lymph nodes from antigen presenting cells, after leaving the node and traveling to the site of tumor tissue, they are unable to infiltrate it [9,11,12]. This situation occurs when a large amounts of VEGF are present in the neoplastic microenvironment, what promotes the formation of fibroblasts, which are a natural barrier of neoplastic cells. VEGF also promotes the neovascularization of neoplastic tissue, while this could appear to promote the penetration of immune cells into the tumor. However, we should remember that the blood vessels growth into the tumor tissue occurs in a quite disorganized and chaotic manner. This situation of resistance is most commonly associated with the occurrence of tumors with immune exclusion, where immune cells are at the periphery or within the stromal tissue [11,12,15].

The unfavorable TME could exclude the immune system outside the tumor. Adenosine and ATP are present in a very low concentrations in extracellular space. However, inflammation, ischemia, or neoplastic processes can lead to the release of ATP through cells membrane, and directly from damaged cells. Extracellular ATP acts as a damage-associated molecular patterns (DAMPs) promoting an immune response. However, during inflammation, extracellular ATP is gradually dephosphorylated by ectonucleotidases (mainly CD39 and CD73), with the consequent formation of adenosine. Adenosine binds to its A1, A2a, A2b and A3 receptors. Stimulation of the A2a receptor inhibits the activity of cytotoxic T lymphocytes and promotes the activity of Treg lymphocytes by increasing the expression of FoxP3. The expression of negative immune checkpoints, including PD-1, CTLA-4 and LAG-3, increases on effector lymphocytes [13,14]. Another substance that eliminates lymphocytes from the tumor area is indoleamine-2,3-dioxygenase (IDO). IDO is an enzyme that metabolizes tryptophan to kynurenine. The production of IDO by cancer cells lowers the level of tryptophan in TME. Tryptophan is an essential amino acid necessary for the proper functioning of lymphocytes. Its absence in TME prevents infiltration of T lymphocytes into the tumor. This results in the formation of tumor with immune exclusion The addition of adenosine or IDO inhibitors to classic ICIs may overcome ICIs’ resistance, which is associated with unfavorable TME [15].

The resistance mechanism, which probably raises the most controversy, is due to the inactivation of immune system cells presented in the tumor tissue. That inhibition can occur on several levels [8,16,17]. One of the most important mechanisms is the activity of myeloid-derived suppressor cells (MDSCs), which are a heterogeneous cells population of myeloid origin that enrich tumor tissue with a very strong immunosuppressive signals. MDSCs consist of myeloid progenitors and immature macrophages, immature granulocytes and immature dendritic cells. In the steady state condition, immature myeloid cells usually lack suppressive activity and are found to be located in the bone marrow, but not in secondary lymphoid organs. In response to various growth factors and cytokines, most notably found within the tumor microenvironment, MDSCs begin to accumulate in lymphoid organs and in tumors and they start to transform into activated state. This is characterized by the increased production of reactive oxygen, nitrogen species as well as arginase. MDSCs have very potent ability to suppress various T-cell functions through direct contact and through the strong immunosuppressive factors that they produced. One of the important pathways involved in the immunosuppressive activity of MDSCs is STAT-1 dependent mechanism. After IFNγ-mediated signaling, which is produced by activated T cells, STAT-1 starts to be activated and, as a consequence, it stimulates the up regulated expression of immune suppressive factors in MDSCs, such as arginase and inducible nitric oxide synthase. In tumor microenvironment, based on the high expression of the granzyme 1 (Gr1) molecule as well as low expression of F4/80, MDSCs can be differentiated from tumor-associated macrophages (TAMs), which are Gr1-negative and F4/80-positive cells. Moreover, large proportion of MDSCs have a granulocytic morphology and possessed the high ability to up-regulated the expression of both arginase and inducible nitric oxide synthase, which is not specific to TAMs [18].

The extremely important immunosuppressive mechanism in tumor microenvironment is the activity of tumor-associated macrophages (TAMs), which are initially attracted into tumors by macrophage chemoattractants (e.g., CCL2, monocyte chemoattractant protein 1 -MCP-1 or by colony-stimulating factor 1 (CSF-1). It is well established that TAMs drive tumor progression and within the tumor microenvironment two completely different functionally subgroups of macrophages are distinct: M1 and M2 cells. M1 macrophages, which are defined as classically activated macrophages, possessed strong anti-cancer properties, they are normally activated during acute inflammation by toll-like receptor (TLR) ligands or by cytokines released mostly by Th1 cells (e.g., IFNγ, TNFα). M1 macrophages produce a high amount of pro-inflammatory cytokines, reactive oxygen species (ROS), and nitric oxide (NO). On the other hand, M2 macrophages, which are also defined as alternative activated state of macrophages, have strong pro-tumoral functions. Under the IL-4, IL-13, IL-10 or TGF-β stimulation, macrophages could convert into M2 phenotype with high ability to subsequently produce anti-inflammatory cytokines (IL-10, TGFβ) that have an inhibitory effect on cytotoxic CD8^+^ T cells. In cancer tissue, TAMs usually exhibit a M2 phenotype and participate to tumor angiogenesis, tumor invasion and metastasis, immunosuppression and cell activation [19].

The inhibition of the immune system also could take place through the direct receptor-receptor interaction. Of note, PD-L1 molecule, which is the best known trail braking activity of PD-1-positive T cells, could be also located on the surface of other immune system cells. Therefore, it should be remembered that not only PD-L1 on tumor cells could inhibit the activity of T lymphocytes [8,9,16,17].

An extremely important role in tumor progression and its resistance to immunotherapy is applied for regulatory T lymphocytes (Treg). In steady state condition, regulatory T cells suppress excessive immune responses to self- and non-self-antigens to maintain immune homeostasis. However, their function in tumor progression is very strongly marked. They are chemoattracted to the tumor microenvironment by the chemokine gradients such as CCR4-CCL17/22, CCR8-CCL1, CCR10-CCL28, and CXCR3-CCL9/10/11. Regulatory T cells (defined as double positive CD4 and CTLA-4 cells) could inhibit costimulatory molecules CD80 and CD86 (expressed by dendritic cells) interaction with CD28 molecules (expressed by T cytotoxic lymphocytes) through CTLA-4 molecules. Moreover, Treg with high expression of CD25 molecules, which is defined as α-chain receptor for IL-2, are mostly responsible for the utilization of IL-2—the cytokine essential for the proper functioning of cytotoxic lymphocytes. In various types of cancer, the high infiltration by regulatory T lymphocytes is associated with poor survival and could predict tumor progression after immune checkpoint inhibitors therapy [20,21,22].

However, as in other immune cell subpopulations, detailed immunophenotyping of Treg has shown that, in secondary lymphoid organs, these cells could differentiate into PD-1-expressing follicular regulatory T cells (TFR). They are characterized as cells positive for surface molecules of CXCR5, GITR-2 and intracellular co-expression of the transcription factors FOXP3 and BCL-6. In terms of their functioning, TFR display higher suppressive capacity when compared with traditional T regulatory cells. Some data indicated that TFR cells are likely to be present in the tertiary lymphoid structures (TLS) of tumors, they also account for a substantial proportion of tumor-infiltrating CD4-positive T cells and could significantly modulate immune responses after ICIs treatment. Therefore, a novel approach to immunotherapy schedule may be the depletion of TFR cells or blocking their activity with anti-CTLA-4 before anti-PD-1 therapy. The effectiveness of this approach has been proven in mouse tumor models and were also associated with better survival outcomes in a large cohort of patients with melanoma [21,22,23].

Finally, the prolonged inflammation in the tumor microenvironment, as well as molecular changes in the tumor cells genome, contribute to the appearance of other inhibitory immune checkpoints on the T lymphocytes surface. The expression of other negative immune checkpoints as VISTA, LAG-3, TIM-3 could be an indicator of exhausted immune cells in TME. On the other hand, they could serve as an additional target for combination immunotherapy as well as for breaking down resistance. These mechanisms described above are observed mainly in the case of acquired or adaptive resistance and are specific for “hot” tumors which are strongly infiltrated by immune cells [8,9,16,17,24].

It should also be kept in mind that the paucity of immune system activity in the tumor microenvironment also results from the ineffective recognition of tumor cell antigens (especially when the function of antigen presenting cells is impaired). The combination therapies based on chemotherapy regiments and ICIs seem to have a chance to overcome this tumor resistance. It is obviously known that anticancer therapies cause immunogenic or inflammatory cell death, which results in breaking cancer cell-driven immunoresistance. An immunogenic apoptosis, and nowadays also pyroptosis, defined as a lytic pro-inflammatory type of programmed cell death, could possesses the ability to activate the immune system. Pyroptosis as an inflammatory cell death seems to have a double and not fully explained role in tumor progression. Pyroptotic tissues could release strong inflammatory mediators, such as HMGB1, which in turns serve as an immune stimulants and can induce the activation of dendritic cells and antitumor T cells. This certainly translates into a higher efficacy of immunotherapy in such patients; however, this thesis should be investigated in prospective clinical trials. On the other hand, some research has indicated that pyroptosis could contribute to the increased hypoxia of neoplastic tumors what is strongly correlated with reduced survival. One possible explanation for this dual effect of pyroptosis is the difference in microenvironment in which the induction of pyroptosis occurs. The chronic inflammation activation of pyroptosis facilitates the tumor progression, while it inhibits tumor growth during acute inflammation. However, the interaction between pyroptosis and antitumor immunity still needs further research [22,23,25].

**Figure 1:**
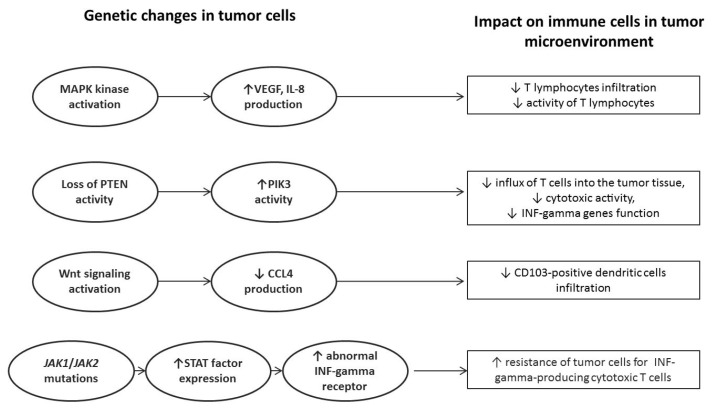
Impact of genetic abnormalities detected in tumor cells on the function of anticancer immune response.

However, it should be absolutely remembered that any resistance to immunotherapy is not due to a single disorder, either in cancer cells or in the immune system [24,26]. Molecular disorders detected in neoplastic tissue have a huge direct impact on the tumor microenvironment and, more precisely, on the presence of individual immune cells and their pro-or anti-tumor activity. The excessive activation of the intracellular MAPK kinase pathway causes increased production of IL-8 and VEGF by tumor cells and, as a consequence, the inhibition of infiltration of T lymphocytes into the tumor tissue. Another example would be a loss of activity of the PTEN protein pathway, resulting in an increased activity of phosphatidylinositol-3-kinase (PI3K). High activity of PI3K affects the reduced influx of T cells into the tumor tissue, reducing their cytotoxic activity and depleting the interferon gamma (INF-γ) genes function. Excessive activation of intracellular Wnt signaling pathways results in impaired or even suppressed production of macrophage inflammatory protein 1 (MIP-1), which belongs to the family of chemotactic cytokines. This chemokine is well known for its chemotactic and proinflammatory effects, and its absence in the neoplastic microenvironment reduces the infiltration of tumor tissue with CD103-positive dendritic cells (DCs). DCs CD103+ contribute significantly to the cytotoxic T lymphocyte response. The last, but very important, genetic disorder is the occurrence of JAK1/JAK2 genes mutations, which translate into excessive activity of STATs family proteins and, consequently, into the appearance of an abnormal form of IFN-gamma receptor on the tumor cell surface [24,25,26]. This is crucial for the resistance of cancer cells to this cytokine, as they become insensitive to the activity of IFN-gamma-producing cytotoxic T cells. This mechanism is one of the main mechanisms of acquired resistance to immunotherapy. Main genetic changes in the tumor genome and their impact on the microenvironment are summarized in Figure 1.

## 3. Genetic Background of Immunotherapy Failure

Over past decades the advancement in high-throughput sequencing technology has increased the numbers of prognostic markers and therapeutic targets [27,28,29]. This development has contributed to understanding the oncogenesis of lung cancer and revolutionized the personalized treatment [27]. To date, tumor PD-L1 expression and tumor mutation burden have emerged as the predictive biomarkers for ICIs in NSCLC; however, both seem to be imperfect tools [27,29]. PD-L1 expression, which states the point of ICIs binding, has various clinically approved cut-off scores and IHC tests that may be impacted by methodological variabilities [28]. Similarly, TMB, which affects the rate of neoantigens production and increases the probability of triggering an adaptive immune response [27,29], may be affected by analytical factors and different sequencing genome coverage and depth applied by whole exome sequencing (WES) or targeted sequencing (TS) approaches [28]. Moreover, TMB cannot predict the immunogenicity driven by synonymous alterations, such as copy number alterations (CNAs) and frameshift indels (small insertions and deletions) that are also considered as a highly immunogenic mutational classes, increasing numbers of neoantigens associated with the sensitivity to ICIs [28,30,31,32].

On the other hand, both PD-L1 and TMB may be affected by intratumor heterogeneity (ITH) that leads to a heterogeneous immune response in distinct populations of cancer cells [28,31]. ITH is often related to discrepancies between different regions within the TME and may be detected at genetic or immunological level [28]. Till now, the association between the predictive value of PD-L1 expression on immune cells distributed in TME and the response to ICIs is very poorly studied in NSCLC patients [31,32]. However, the increased ITH of neoantigens may elevate the expression of cells’ subclone with poor immunogenicity, impairing the ICIs effectiveness [24,33]. In that context, for better treatment, a deeper analysis of dependencies between PD-L1 expression, TMB and ITH, and real prognostic and predictive values of well-known and available factors seem to be vital to determine.

Despite the limitations, both PD-L1 and TMB are currently used to determine the potential sensitivity of NSCLC to ICIs; however, only 15–20% of patients exhibit a long-term response to ICIs [24,31,32]. Within the first two months of ICIs’ administration, 40–50% of patients experience the failure of treatment that is clinically manifested by progression or even hyperprogression of the primary tumor or metastases [8,34,35]. This intrinsic resistance is regulated by different aberrations in oncogenes and suppressor genes’ that affect the immune response by amending cytokines’ profile and immune cells’ composition rendering tumor cells resistance or sensitivity to ICIs [18,29].

*RET* rearrangements [36] and *HER2* mutations [37] are associated with a low PD-L1 expression, while *EGFR* activating mutations [38], *ALK* rearrangements [39], *ROS1* rearrangements [37] and *MET* exon 14 skipping mutations [40] relate with a high PD-L1 expression simultaneously decreasing tumor mutations burden and the level of tumor infiltrating lymphocytes (TILs) that result in limited response to ICIs [24,31]. Especially, *EGFR* activating mutations shape a neutral immune environment by modulation of immunosuppressive cells and cytokines heaving the impact on T-cell exhaustion and reduction of cytotoxic lymphocytes [38,39,40]. Moreover, the downstream of *EGFR* mutated pathways, such as MAPK, PI3K/AKT and JAK/STAT, negatively affect immune regulation [24]. In contrast *BRAF*-mutated NSCLC patients seems to show a higher PD-L1 expression with increased sensitivity to ICIs; however, it has not been confirmed well enough in clinic [31,37,41]. Likewise, NSCLC patients with overlapping of *KRAS* and *STK11/LKB1* mutations’ have showed a higher clinical sensitivity for ICIs due to epigenetic inhibition of stimulator of INF genes (*STING*) [24,29,42]. On the other hand, loss of *STK11/LKB1* overlapping with oncogenic *KRAS* mutation is associated with a high level of IL-6, resulting in increased neutrophils recruitment and decreased T-cells infiltration [24,31,42]. Moreover, *STK11* mutations often coexist with *KEAP1* mutations that relates to cellular resistance for oxidative stress [43,44]. Likewise, co-occurrence of *KEAP1* mutation and *PTEN* inactivation is characterized as an indicator of a “cold” tumor [24,43,44]. Afterwards, the alterations of the AKT/PTEN pathway may suppress the PI3K signaling and decrease TILs activity, leading to the primary resistance to immunotherapy despite high PD-L1 expression [31,35]. Subsequently, changes in the copy number of *MDM2*, *MDM4*, *CCND1*, *FGF3*, *FGF4* and *FGF19* genes, as well as overexpression of genes related to epithelial–mesenchymal transformation (*AXL*, *WNT5A*, *ROR2*, *TWIST2*, *FAP*, *TAGLN* genes), vascular endothelial growth factor-dependent signaling pathway (*IL-10*, *VEGFA*, *VEGFC* genes) and macrophage chemotactic factor (*CCL2*, *CCL7*, *CCL8*, *CCL13* genes) are considered as the other intrinsic factors of resistance to ICIs [29,32,34]. Till now, 26 transcriptional signatures related to many cellular processes have been referred to as the indicators of innate anti-PD-1 resistance (IPRES) in metastatic melanoma [45]. However, the IPRES value of 700 genes’ was proposed by in silico gene ontology evaluation of the TCGA (The Cancer Genome Atlas) database in the subset of tumors. However, its diagnostic value for evaluation of ICIs efficiency is limited and should be verified in clinical trials [31].

The acquired resistance to ICIs develops in 25–35% of NSCLC within the first 12 months of treatment, and it is driven by a dynamic regulation of the immune microenvironment affecting the interaction between immune and cancer cells [8,34]. The pro-immune escaping nature may be induced by loss of neoantigens, production of immunomodulators and selection of subclones harboring the driver mutations [8,35]. However, all of this mechanisms may overlap and effect on each other. The loss of neoantigens may be related to both clonal selection (exhaustion of neoantigens) and acquisition of CNAs (deletions of *PTEN*, *C17orf78*, *HSD17B1* and *WNK4* genes) [34]. A favorable impact on clinical outcome requires the clonal immunogenic neoantigens presented at 100% of the tumor cells [31,33]. However, TILs activated by ICIs primarily recognize immunogenic neoantigens and decrease their expression sculpturing the non-immunogenic subclone of neoantigens that do not elicit an effective antitumor response [8,30,33]. Moreover, in immune selection process clones of tumor cells that do not express neoantigens can proliferate constituting another escape route [31,32,33]. In that way, even small primary ITH may selectively pressure the independent mechanisms of immune evasion.

TME immunomodulation is also related to mutations and polymorphisms in the genes of the interferon signaling pathway (*IFNGR1*, *IFNGR2*, *JAK1*, *JAK2*, *IRF1* genes) and pro-angiogenic factors secreted by macrophages (*VEGF*, *EGF*, *MMP* genes), factors inhibiting the activity of TILs (*ARG1*, *PGE2*, *TGF-β* genes) and chemokines (*CCL5*, *CCL17*, *CCL22*, *CXCL8*, *CXCL12* genes) [31,32,34]. Additionally, dysfunctional mutations in *JAK1/2* and *B2M* genes have been described as the factors of secondary resistance to ICIs that may respectively affect the INF production by TILs or impair cell surface expression of MHC class I molecules [24,29,31,32,34]. Likewise, the resistance to immunotherapy may be acquired as a result of immune cells depletion within the tumor, as well as overexpression of genes encoding alternative immune checkpoint receptors (TIM-3, LAG-3, BTLA, TIGIT and VISTA) that inhibit the immune response and are associated with adaptive resistance to anti-PD-1 therapy in NSCLC patients [29,46]. On the other hand, the alternative receptors are considered as potential therapeutic targets for the next generations of ICIs [46].

## 4. A Clinical Approach to Immunotherapy Resistance

Various clinical trials are currently exploring the possibilities of overcoming resistance to immunotherapy. Clinicaltrial.gov (accessed on 11 July 2021) was searched for upcoming data and trials. The following search terms were used: immunotherapy resistance and lung cancer.

Six interesting studies were found among the clinical trials devoted to new treatment options for patients with resistance to ICIs associated with genetic abnormalities. (Table 1). These studies focused on genetic factors that may be predictive of response to immunotherapy. Chromosomal instability (CIN) is one of the most important heterogeneity of cancer genomes. CIN accelerates phenotypic adaptation under selective pressures encountered during tumor evolution and therapy. CIN has important impact on development of anticancer drug resistance, causing treatment failure and disease recurrence [47]. Jamal-Hanjani et al. studied intratumor heterogeneity in relation to clinical outcome in early-stage NSCLC. They observed elevated genes copy number heterogeneity what was related with an increased risk of recurrence or death [48]. In the study NCT04203095, researchers evaluated dynamic CIN continuously monitored in the blood of patients with lung cancer treated with ICIs. Ultrasensitive chromosomal aneuploidy detection (UCAD) was used to establish a new molecular immune resistance evaluation index. Further, the correlation between the evolution of tumor clonality and ICIs resistance in patients during treatment was analyzed based on the results of dynamic CIN detection. This study enabled better understanding and overcoming the resistance mechanism of immunotherapy.

The approach to research with genetic testing in predicting immunotherapy resistance is diverse. Investigators conducting study NCT04300062 aimed to collect tumor tissue at the moment of progression during ICIs therapy, in order to insure a later study on molecular mechanism involving the progression of NSCLC and SCLC.

In the study NCT03512847 predictive molecular profiles for immunotherapy or chemotherapy resistance were found by comparison of treatment outcome for patients with specific molecular characteristics. Differences in molecular profiles pre- and post-treatment may reveal resistance mechanisms to treatment. The authors of this study wrote only a paper about clinical relevance of re-biopsy in progressive, advanced NSCLC after first-line treatment. The conclusions have been obtained from the research to date about changes in percentage of tumor cells with PD-L1 expression. Changes in percentage of tumor cells with PD-L1 expression was observed in 33% of patients (*n* = 15) and 17% patients (*n* = 8) had potentially clinically relevant changes. A significantly higher chance of change in PD-L1 expression was observed in patients who received chemotherapy [49].

In study NCT04807114, investigators collected tumor biopsies from advanced NSCLC patients before start of treatment with immune checkpoint inhibitors to characterize the tumor microenvironment. They also profiled the immune composition of peripheral blood. A second aim of this study was to characterize the immune cell composition of bronchoalveolar lavage (BAL) fluid from cancer patients treated with ICIs to predict development of pneumonitis.

DARWIN II (NCT02314481) is an exploratory phase II study examining the role of intratumor heterogeneity and presence of neoantigens on the effectiveness of anti-PD-L1 immunotherapy in *EGFR* or *HER2* mutated NSCLC patients with relapsed disease after afatinib therapy. Researchers examined whether intratumor heterogeneity (clonal vs. subclonal actionable mutations) is associated with progression-free survival (PFS) in patients treated with atezolizumab or molecularly targeted therapy.

NCT04405661 is observational study, which evaluated the differences of genes mutations and immune microenvironment in NSCLC patients with different response to ICIs therapy.

Information on ongoing clinical trials on the genetic background of resistance to immunotherapy is summarized in Table 1.

Researchers are looking for different options of combination therapy to break down resistance to immunotherapy. Biological rationale supports the potentiality of combining ICIs with a number of non-chemotherapy agents. Various options are being tested, one of which is the use of anti-angiogenic drugs with ICIs. A key point in tumor development is the ability to induce angiogenesis. The process of angiogenesis is promoted by inflammatory mediators, which in turn can affect the immune microenvironment. Anti-angiogenic agents can induce anti-angiogenesis by targeting vascular endothelial growth factor or inhibiting multiple small molecules involved in angiogenic and proliferative pathways such as tyrosine kinases of platelet-derived growth factor receptors (PDGFRs) and fibroblast growth factor receptors (FGFRs). Anti-angiogenic substances can stimulate the immune system, and the other way immunotherapy can also have anti-angiogenic effects. These two types of therapy can act synergistically on the inhibition of tumor growth [50].

Three ongoing studies examined the effectiveness of combination of anti-angiogenic drugs and immunotherapy: NCT04782622, NCT04691388, NCT04670107. However, there are no results available yet. Furthermore, the preliminary results of phase III IMpower 150 study showed an acceptable toxicity profile and encouraging antitumor activity of ICIs combined with anti-angiogenic agents in patients with advanced NSCLC. This clinical trial was concerned about treatment with atezolizumab plus bevacizumab plus chemotherapy as first-line treatment in patients with metastatic nonsquamous NSCLC. The outcomes showed that this combination therapy significantly improved PFS (8.4 months vs. 6.8 months; HR = 0.59; 95% CI = 0.50–0.69) and OS (19.8 months vs. 14.9 months; HR = 0.76; 95% CI = 0.63–0.93) compared to therapy without atezolizumab [51].

Clinical studies on the combination of ICIs with chemotherapy have been conducted many times. Dafni et al. conducted a meta-analysis comparing different ICIs combinations in first-line treatment of advanced NSCLC. They received a very significant results. First of all, chemotherapy has less efficacy in comparison to any ICIs and chemotherapy combination. Moreover, pembrolizumab or atezolizumab with chemotherapy are the best treatments in the overall cohort. Results are coherent for patients with different histopathological diagnosis or PD-L1 expression [52].

PIONEER clinical study (NCT03833440) investigated how to overcome resistance to ICIs monotherapy or in combination with platinum-based chemotherapy. Intervention model description included four arms of drug combinations. Arm A used combination of durvalumab and inhibitor of NKG2A (CD94)—monalizumab (IPH2201) to target a PD-L1 co-inhibitory pathway. Arm B was combination of durvalumab anti-CD73 antibody—MEDI9447 to target limitations of antitumor T-cell immunity caused by adenosine receptor signaling. Arm C used of durvalumab plus ATR inhibitor—AZD6738 to potentially enhance anti-tumor T-cell responses. Arm D was a standard third or fourth-line chemotherapy (docetaxel).

The results of a phase I CheckMate 012 study evaluated combination therapy with nivolumab and ipilimumab in patients with advanced NSCLC (NCT01454102). In NSCLC, first-line nivolumab plus ipilimumab had a tolerable safety profile and showed encouraging clinical activity, characterized by a high response rate and durable response. These results brought about the CheckMate 227 study and CheckMate 9LA study. CheckMate 227 clinical trial compared this regimen to standard first-line chemotherapy, nivolumab monotherapy or combination therapy with chemotherapy and nivolumab for patients with advanced NSCLC. This study showed that first-line treatment with nivolumab plus ipilimumab resulted in a longer duration of overall survival than chemotherapy in NSCLC patients independent of the PD-L1 expression [53]. CheckMate 9LA indicated that nivolumab plus ipilimumab with two cycles of chemotherapy provided a significant improvement in overall survival versus chemotherapy alone, and had a favorable risk-benefit profile [54]. Thanks to the beneficial results of the above study, investigators propose an idea to addition of ipilimumab to nivolumab, after primary resistance to anti-PD-1 therapy, which could cause radiographic tumor regression. The study NCT03262779 included NSCLC patients who had progression after anti-PD-1 therapy without initial response to such therapy (primary resistance). Moreover, a smaller cohort of patients with acquired resistance to anti-PD-1 therapy (progression after initial response) was also enrolled to this study.

Another idea is combining atezolizumab and cobimetinib (MEK inhibitor) in treatment of NSCLC patients with cancer spread to other places in the body (metastases), or has recurrent disease, or does not respond to treatment (refractory disease) (NCT03600701). MEK inhibition may promote accumulation and survival of intratumoral tumor-specific T cells and can synergize with immune checkpoint inhibition. Hellmann et al. used atezolizumab and cobimetinib in immunotherapy-naive patients with solid tumors. Atezolizumab with cobimetinib had manageable safety and clinical activity regardless of *KRAS* and *BRAF* genes status. Confirmed responses were observed in seven of 84 patients (8%) with metastatic colorectal cancer (mCRC) (six responders were microsatellite stable or with low MSI and one patient had high MSI), nine of 22 patients (41%) with melanoma, and five of 28 patients (18%) with NSCLC. Although synergistic activity was seen in mCRC, this was not confirmed in a phase III study [55].

Cichocki et al. developed a method of producing natural killer cells from induced pluripotent stem cells (iPSC). iPSC-derived NK cells (iNK) produce inflammatory cytokines and exert strong cytotoxicity against of hematological and solid tumors. Moreover, they showed that iNK cells recruit T cells and cooperate with T cells and the anti-PD-1 antibody additionally enhancing inflammatory cytokine production and tumor destruction [56]. NCT03841110 clinical study used iNK cells (FT500), which may connect innate and adaptive immunity and has the potential to overcome multiple mechanisms of ICIs resistance in advanced solid tumors.

The results of clinical trials using different concepts of combination therapies to convert resistance to classical ICIs are presented in Table 2.

Idea of combining immunotherapy with radiation therapy is a worth considering issue. Radiation therapy (RT) causes the death of cancer cells, which can increase the level of cancer antigen in the bloodstream, promoting recognition of cancer cells by the immune system and activation against cancer cells. This is evidenced by the abscopal effect. The abscopal effect is the ability of localized radiation to induce an immunological antitumor response in distant metastases that were not subjected to targeted radiotherapy [57]. Radiation can also reverse immunotherapy resistance by reducing lymphocyte depletion. Several studies are underway looking at the combination of immunotherapy with radiation therapy. The most important of these studies was the PACIFIC clinical trial, in which durvalumab was used in consolidation therapy after successful concurrent chemoradiotherapy in patients with locally advanced NSCLC. Durvalumab compared to placebo significantly prolonged overall survival of patients (47.5 months vs. 29.1 months). The results of this study resulted in the registration of durvalumab in this indication [58]. RTOG (Radiation Therapy Oncology Group) conducted one of the first phase III clinical trials for the application radiotherapy and immunotherapy in stage III NSCLC patients (NCT02768558). In RTOG study patients received thoracic radiation, cisplatin and etoposide followed by nivolumab or placebo given every two weeks for one year. This trial showed insight into this therapeutic approach, including OS and PFS endpoints, patient-reported outcomes, quality of life, and relevant biomarker studies [59]. Locally advanced NSCLC patients treated with concurrent chemoradiation may represent the optimal setting for anti-PD1 immune checkpoint inhibitors therapy. Another study in which the effectiveness of the combination of chemotherapy, radiotherapy and immunotherapy was assessed was NIRVANA—Lung clinical trial (NCT03774732). This study involved concurrent chemoradiotherapy and pembrolizumab in advanced NSCLC patients.

Two clinical trials were designed to convert resistance to classical ICIs. The NCT04472949 clinical trial enrolled extensive-stage SCLC patients. The main purpose of this study was to evaluate the efficacy of thoracic radiotherapy combined with maintenance durvalumab after first-line therapy with carboplatin and etoposide plus durvalumab. The primary endpoint in this study was progression-free survival, and the study was scheduled to end in the spring of 2021. The secondary objective was to evaluate the safety of radiotherapy combined with maintenance durvalumab in SCLC patients after chemoimmunotherapy. The second notable study is NCT03224871 conducted in metastatic NSCLC who have progressed after checkpoint inhibition. The investigators hypothesized that combination of radiotherapy and intralesional injection of interleukin-2 (IL-2), involved in the activation of leukocytes, may defeat resistance to ICIs and offer significant clinical benefits. Similar ideas with the use of IL-2 appeared before. Topalian et al. suggested possible responses to nivolumab after prior IL-2 treatment [60].

The above-mentioned studies trying to validate these new treatment options are still ongoing. We still have to wait for the final results of the cited studies. However, they can significantly change current treatment regimens.

There are still many options for overcoming drug resistance that could be discussed such as bi-specific antibodies (BsAbs). BsAbs recognizing two different epitopes may be a potentially effective method of immunotherapy and they are worth mentioning. BsAbs are usually divided into two types: IgG-like and non-IgG-like. Functioning pathways may be quite flexible, some BsAbs can play the role of immune cell connector, connecting immune cells to tumor cells and enabling immune cells to exert their killing effect. BsAbs can also target tumor antigens, blocking dual signaling pathways. The first bispecific antibody, Blinatumomab, targeting the CD3 epsilon domain of TCRs and CD19, is used to treat acute lymphoblastic leukemia [61,62].

## 5. Conclusions

Immune checkpoint inhibitors have changed the management of lung cancer patients. Despite high hopes for immunotherapy, a lower than expected percentage of lung cancer patients achieve sustained anti-cancer efficacy from ICIs therapy. Identifying predictive biomarkers for the indication of patients who are likely to benefit from the ICIs treatment is crucial. Improving combination therapies and defining optimal strategies for advanced lung cancer will be an opportunity to overcome ICIs resistance and wider use of anti-PD-1, anti- PD-L1 and anti-CTLA-4 antibodies. Studies to evaluate TMB, molecular signatures and the tumor microenvironment as biomarkers for predicting ICIs efficacy have been conducted, but they are rarely used in clinical practice. Moreover, epigenetic alterations have an effect on the effectiveness of immunotherapy. Moreover, research on combined treatments in patients with epigenetic-driven cancers are challenging. The results of the presented research on combined therapies as well as the results of the conducted molecular studies may be interesting and relevant, and also may affect new treatment strategies.

## Figures and Tables

**Table 1:** Ongoing clinical trials investigating the genetic background of resistance to immunotherapy in lung cancer patients.

Clinical Trial ID	Primary Outcome Measures	Intervention/Treatment
NCT04203095	Correlation analysis between the ICIs resistance and CIN.	Diagnostic test: the level CIN in plasma cfDNA. Anti-PD-1 antibody treatment.
NCT04300062	Molecular mechanism involving the progression of NSCLC and SCLC	Re-biopsy at the moment of cancer progression. Immunotherapy.
NCT03512847	Concordance between specific gene profiles and treatment outcomes. Differences in molecular profiles pre- and post-treatment. Quantification of cfDNA during treatment linked to treatment outcome.	Comprehensive molecular profiling and whole exome sequencing (WES) of cfDNA. Immunotherapy/chemotherapy.
NCT04807114	Identification and comparison the percentages of immune cell subtypes present in tumors from responding vs. non-responding the patients before start of ICIs therapy.	Tumor biopsies from patients before start of treatment. Anti-PD-1 monotherapy or combination of anti-PD-1 therapy and chemotherapy.
5	Identification of actionable mutations and intratumor heterogeneity in NSCLC patients with *EGFR* or *HER2* genes mutations and relapse after afatinib treatment. Qualification to different treatment regimens and measurement of therapy effectiveness (ORR, PFS)	Biopsy sample at the time of NSCLC relapse. Arm 1: Patients without an actionable mutation—atezolizumab monotherapy or in combination with chemotherapy; Arm 2: Patients with *BRAF* V600 mutation—vemurafenib; Arm 3: Patients with *ALK* or *RET* genes rearrangement—alectinib; Arm 4: *HER2* gene amplification—trastuzumab emtansine.
5	Correlations between specific genes mutations, immune microenvironment and ORR as well as PFS in patients received immunotherapy.	Sequencing of DNA from FFPE tissue and whole blood. IHC analysis of immune microenvironment. Anti-PD-1 or anti-PD-L1 antibody treatment.

**Table 2:** Clinical trials on drug combinations and breaking down resistance to immunotherapy.

Clinical Trial ID	Primary Endpoint	Treatment
NCT04782622	ORR and PFS	apatinib + camrelizumab
NCT04691388	PFS	Anlotinib + sinidilizumab
NCT04670107	PFS and OS AE (adverse event caused by the combination)	anlotinib in combination with immune checkpoint inhibitors
NCT03833440	The 12-week disease control rate	Arm A: durvalumab + monalizumab (IPH2201), Arm B: durvalumab + oleclumab (MEDI9447), Arm C: durvalumab + ceralasertib (AZD6738), Arm D: standard of third or fourth-line chemotherapy (docetaxel)
NCT03262779	ORR	nivolumab + ipilimumab
NCT03600701	ORR	atezolizumab + cobimetinib
NCT03841110	Maximum tolerated dose	FT500 + cyclophosphamide + fludarabine FT500 + nivolumab + cyclophosphamide + fludarabine FT500 + pembrolizumab + cyclophosphamide + fludarabine FT500 + atezolizumab + cyclophosphamide + fludarabine FT500 + IL-2 (proleukin, aldesleukin) + nivolumab + cyclophosphamide + fludarabine FT500 + IL-2 (proleukin, aldesleukin) + pembrolizumab + cyclophosphamide + fludarabine FT500 + IL-2 (proleukin, aldesleukin) + atezolizumab + cyclophosphamide + fludarabine

## Data Availability

Not applicable.
